# Reduced Syncytin-1 Expression Levels in Placental Syndromes Correlates with Epigenetic Hypermethylation of the ERVW-1 Promoter Region

**DOI:** 10.1371/journal.pone.0056145

**Published:** 2013-02-14

**Authors:** Matthias Ruebner, Pamela L. Strissel, Arif B. Ekici, Elisabeth Stiegler, Ulf Dammer, Tamme W. Goecke, Florian Faschingbauer, Fabian B. Fahlbusch, Matthias W. Beckmann, Reiner Strick

**Affiliations:** 1 University-Clinic Erlangen, Department of Gynaecology and Obstetrics, Laboratory for Molecular Medicine, Erlangen, Germany; 2 University-Clinic Erlangen, Institute of Human Genetics, Erlangen, Germany; 3 University-Clinic Erlangen, Department of Paediatrics and Adolescent Medicine, Erlangen, Germany; VU University Medical Center, The Netherlands

## Abstract

Terminal differentiation of villous cytotrophoblasts (CT) ends in formation of the multinucleated syncytiotrophoblast representing the fetal-maternal interface. Aberrations during this cell-fusion process are associated with Intrauterine Growth Restriction (IUGR), Preeclampsia (PE) and High Elevated Liver and Low Platelets (HELLP) Syndrome. Syncytin-1, the envelope gene of the human Endogenous Retrovirus ERVW-1, is one of the most important genes involved in cell-fusion and showed decreased gene expression during these pathological pregnancies. The aim of this study was to determine the methylation pattern of the entire promoter of ERVW-1 and to correlate these findings with the expression profile of Syncytin-1 in the placental syndromes. 14 isolated villous cytotrophoblasts from control (n = 3), IUGR (n = 3), PE (n = 3), PE/IUGR (n = 3) and HELLP/IUGR (n = 2) placentae were used to determine the mean methylation level (ML) for the ERVW-1 promoter region. ML rose significantly from 29% in control CTs to 49% in IUGR, 53% in PE, 47% in PE/IUGR and 64% in HELLP/IUGR indicating an epigenetic down-regulation of Syncytin-1 by promoter hypermethylation. DNA demethylation of the trophoblast like cell lines BeWo, JEG-3 and JAR with 5-AZA-2′desoxycytidine (AZA) showed an increased Syncytin-1 expression and fusion ability in all cell lines. Promoter activity of the 5′LTR could be inhibited by hypermethylation 42-fold using a luciferase based reporter-gene assay. Finally overexpression of the methyltransferases DNMT3a and LSH could be responsible for a decreased Syncytin-1 expression by promoter hypermethylation of ERVW-1. Our study linked decreased Syncytin-1 expression to an epigenetic hypermethylation of the entire promoter of ERVW-1. Based on our findings we are predicting a broad aberrant epigenetic DNA-methylation pattern in pathological placentae affecting placentogenesis, but also the development of the fetus and the mother during pregnancy.

## Introduction

Epigenetics comprises changes in gene expression, without changing the DNA sequence. These effects are mediated by covalent attachment of chemical groups to DNA and its associated proteins and histones. Epigenetic marks are fixed after the cell has differentiated. In developmental stages as well as in some tumours, a broad epigenetic reprogramming takes place, which results to removing or changing of epigenetic marks [1], [2]. In humans the methylation pattern of CpG-dinucleotides gives information about the activity of affected genes [3]. Hypermethylation of CpGs usually results in an inactivation of chromatin regions [4]. Responsible for placing epigenetic marks are *DNA-methyltransferases* (DNMT) such as DNMT1, DNMT3a and 3b [1]. The *lymphoid-specific helicase* (LSH) could modulate CpG-methylation and is also involved in the de-novo methylation process [5], [6]. Proteins which recognize and bind methylated CpGs by a M*ethyl-CpG-Binding Domain* (MBD) are MBD1-4 and the *methyl CpG-binding protein 2* (MeCP2) [7]. Bound MeCP2 e.g. mediates gene silencing by a *Transcription Repression Domain* (TRD) [8]. The corepressor *mSin3A* can bind to TRDs and recruit *histone deacetylases* (HDAC) which are responsible for gene inactivation by chromatin condensation [9].

During human pregnancy multiple factors are required for the development of a fetus. Formation of a normal placental morphology is one of the key factors involved in this process. During early placentogenesis, when the blastocyst implants into maternal endometrium, *villous cytotrophoblasts* (VCT) fuse to form the multinucleated *syncytiotrophoblast* (SCT) [10] representing the primary feto-maternal barrier for exchange of nutrients, gas and waste products [11]. Pregnancy-associated diseases could be linked to alterations of this placental morphology. *Intrauterine Growth Restriction* (IUGR) has an incidence from 4% to 7% of all live births and is one of the major perinatal problems causing morbidity and mortality of mother and fetus [12], [13]. The surface area of chorionic villi from IUGR placentae (∼8.2 m^2^) compared to control placentae (∼10 m^2^) resulted in a smaller fetal-maternal membrane [14]. In addition, IUGR placentae showed an abnormal cellular development of trophoblasts, like lower amounts of CTs, reduced cell fusion (nuclei per mm SCT) and a higher apoptosis rate [14]–[16]. *Preeclampsia* (PE) is characterised by maternal hypertension and increased urinary protein secretion [17]. PE affects approximately 6% of all pregnancies and causes 15–20% of maternal deaths in developed countries. Furthermore PE results in up to 13% stillbirths and 20% of early neonates in some areas of the world [18], [19]. The *Hemolysis Elevated Liver Enzymes and Low Platelets* (HELLP) - syndrome is also originating from abnormal placentogenesis due to diminished function and can occur alone or in combination with IUGR [20]. The HELLP syndrome involve 10–14% of all pregnancies complicated by PE [21]. These patients suffer under acute renal failure, pulmonary oedema, abruption placentae and intracranial bleeding and are responsible for the high maternal and fetal morbidity [22], [23].

*Human endogenous retroviruses* (ERVs) originate from retroviral infections into the germ line millions of years ago and make up to 8% of the human genome. ERVs belong to the class of retroelements possessing the three viral genes *group-specific antigen* (gag), *polymerase* (pol) and *envelope* (env) flanked by two *long terminal repeats* (LTR) [24]–[26]. Deletions and mutations resulted in inactivation of most of the 400,000 ERV copies, but some sustained an intact open reading frame and are able to express ERV sequences on RNA and protein level [25], [27]–[29]. In the majority of cases ERVs will be expressed tissue specific [30]. In human placentae some ERV env genes play important roles. The placenta-specific ERVW-1 env gene Syncytin-1 is essential for cell fusion of VCT to the multinucleated SCT in human placentae [31], [32]. Recently Syncytin-1 expression was found in leukaemia and lymphoma cell lines and blood samples [33], but also in other tumours like colorectal, breast and endometrial cancer [34]–[36]. The promoter region of ERVW-1 comprises the 5′LTR of the provirus and the *upstream regulatory region* (URE) with the *trophoblast specific enhancer* (TSE) [37], [38] where many factors can regulate Syncytin-1 expression. Forskolin, an activator of the adenylatcyclase raising the cAMP level in cytosol [39], increased the fusion index in trophoblast-like cell lines JAR, BeWo and cultured primary trophoblasts. The binding region of phosphorylated CREB (cAMP response element-binding protein) is located in a 122 bp region within the U3 region of the 5′LTR [38], [40]. Hypoxia led to a decreased Syncytin-1 expression and had negative effects on the cell fusion ability in BeWo cells [41], [42]. The functional process of cell fusion of VCT and other cell types is not understood in detail. Two other ERV env proteins Syncytin-2 (ERVFRD-1) and Syncytin-3 (HERV-P(b)) were shown to regulate cell fusion in vitro and implicated in VCT fusion [16], [43] as well as other genes like connexin 43 [44].

Examples of epigenetic regulation of ERV expression in humans have been shown. Hypomethylation of the ERV-K promoter region in germ cell tumours led to an overexpression of this gene in contrast to the cell line Tera-1, where hypermethylation correlated with inactivation of ERV-K [45], [46]. Furthermore, the LTR of ERVE-1 was hypomethylated in placental cells whereas it was hypermethylated in blood cells [47]. Matousková et al. (2006) could show that the first five CpGs within the 5′LTR of ERVW-1 were also differentially methylated in various tissues. An inactivation of the Syncytin-1 expression by hypermethylation of the 5′LTR appeared in non-placental tissues, whereas in placentae and in BeWo cells these five CpGs were hypomethylated [48]. During pregnancy the 5′LTR of ERVW-1 showed a stage specific methylation pattern in VCT. In the first trimester the 5′LTR promoter region showed no CpG-methylation then the methylation of the CpGs rose from 8.3% in the second trimester to 30% in term VCTs [49]. These findings support an epigenetic reprogramming during placentogenesis. The goal of this project was to unravel, if reduced expression of Syncytin-1 in PE, HELLP and IUGR cultured isolated trophoblasts was due to hypermethylation of the promoter region of ERVW-1 and if an aberrant expression of DNA-methyltransferases could be responsible for these changes.

## Materials and Methods

### Ethics Statement

All participants gave their written informed consent with the approval by the Ethics Committee of the University of Erlangen-Nuremberg.

### Patient and Tissue Collective

A total of 14 human placentae were obtained from controls, PE, IUGR, PE/IUGR and HELLP/IUGR patients with no other clinical diseases, like cancer and diabetes, after elective Caesarean section. From all patients a written consent was obtained. The clinical data of the control cohort and patients are presented in Table 1. A biopsy was obtained near the cord from every placenta, snap frozen in liquid nitrogen and stored at −80°C until further use or were used for VCT fractionations (see below). The diagnosis of IUGR, PE and HELLP was based on general accepted criteria previously described in Langbein et al. (2008) [50].

**Table 1:** Clinical data.

	control n = 3		IUGR n = 3		PE n = 3		PE/IUGR n = 3		HELLP/IUGR n = 2	
	mean	sem	mean	sem	mean	sem	mean	sem	mean	sem
**gestational age**	37.63	±0.18	36.33	±2.33	36,00	±1.67	35.67	±2.96	36,00	±1.68
**gravida**	1.57	±0.3	1.33	±0.33	1.50	±0.34	1.33	±0.33	1.25	±0.30
**para**	0.14	±0.14	0.33	±0.33	0.33	±0.21	0.33	±0.33	0,00	±0.00
**blood pressure**	119.38	±3.83	136.67	±6.67	**168.33**	±4.94	**180,00**	±12.58	**177.5**	±6.29
	70,00	±3.66	78.67	±6.84	**104.17**	±2.01	**108,00**	±11.79	**112.5**	±4.79
**protein**	n.d.		n.d.		**7,111.17**	±2,683.76	**4,360.67**	±2,941.75	**6,332.72**	±175.66
**GOT**	n.d.		n.d.		87,00	±48.78	69.33	±37.84	**489.5**	±127.22
**GPT**	n.d.		n.d.		76.83	±57.92	62,00	±48.00	**357.75**	±79.64
**LDH**	n.d.		n.d.		267,00	±33.09	277.33	±26.01	**892.75**	±150.08
**platelets**	245.67	±77.45	215,00	±33.00	227,00	±23.18	228,00	±33.42	**93.33**	±5.67
										
**weight child**	3,197.5	±203.42	**1,743.33**	±326.82	2,601.67	±417.73	**2,208.67**	±899.59	**2,132.5**	±156.06
**height child**	49.63	±0.84	**40.33**	±3.18	46.73	±2.46	**42.33**	±6.12	**45.25**	±1.6

GOT: Glutamate-Oxaloacetate-Transaminase; GPT: Glutamate-Pyruvate-Transaminase; LDH: Lactate dehydrogenase;

Significant changes marked in bold.

### Fractionation and Cultivation of Cytotrophoblasts

Human CTs were isolated using the trypsin-DNase-dispase-collagenase-hyaluronidase/percoll method [16], [50]–[53] from 3 independent control, 3 IUGR, 3 PE, 3 PE/IUGR and 2 HELLP/IUGR placentae. For quality control see Ruebner et al. [16]. VCTs were seeded at 300,000 viable cells/cm^2^ in a humidified 5% CO_2_ environment at 37°C and cultivated for 3 days. Fractured syncytial cellular fragments, non-adherent cells and debris were removed initially after 4 hr and then every 24 hr with a change of media [54].

### Cell Culture of Trophoblast-like Cell Lines

The trophoblast-like cell lines BeWo (CCL-98), JEG-3 and JAR (all ATCC), derived from choriocarcinomas, were cultured under following conditions: BeWo was grown in RPMI 1640 media (Sigma) supplemented with 10% FCS, 10 mM Hepes, 2 mM L-Glutamin and 0.1 mM NEAA; JEG-3 was grown in Minimum Essential Media (MEM media from Sigma) supplemented with 10% FCS, 10 mM Hepes, 2 mM L-Glutamin, 0.1 mM NEAA and 1 mM Na-pyruvate; JAR was grown in RPMI 1640 media (Sigma) supplemented with 10% FCS, 10 mM Hepes, 2 mM L-Glutamin and 0.1 mM NEAA. Isolated CTs were cultured in DMEM media (Sigma) supplemented with 10% FCS, 10 mM Hepes, 2 mM L-Glutamin, 0.1 mM NEAA and 100 µg/ml Penicillin and Streptomycin (Sigma). For demethylation experiments cell lines were treated with 5-AZA-2′Deoxycytidin (AZA) and Trichostatin A (TSA) for 3 days with following concentrations: BeWo 3.5 µM AZA, 60 nM TSA; JEG-3 1.0 µM AZA, 20 nM TSA; JAR 0.5 µM AZA, 20 nM TSA. For each cell line drug concentration was tested individually. Cell lines were treated with each drug alone or together in a minimum of 4 independent experiments.

### RNA Extraction and cDNA Synthesis

Total RNA was extracted from 50–100 mg of frozen placental tissues according to Strick et al. and Langbein et al. [36], [50]. For expression analysis, RNA was pre-treated with DNase I (Sigma-Aldrich, Germany) and cDNA was generated with the High Capacity cDNA Kit (Applied Biosystems, Germany) in a thermal cycler (ABI2720) for 2 hr at 37°C.

### Absolute (qPCR) and Semi-quantitative (sqPCR) Real Time PCR

QPCR with specific primers was used to quantitate Syncytin-1 (40 ng cDNA/well) with SYBR-green technology (for primers and standard curve see Ruebner et al. [16]). Amplification of 18S-rRNA was used for normalization of different samples using 1 ng cDNA/well. Analysis of DNMT1, 3a, 3b, MBD1-4, MeCP2 and LSH (primers see Table 2) were performed by SYBR-green based sqPCR using trophoblasts with 50 ng cDNA/well. Co-amplification of 18S-rRNA and one control cDNA as internal control were used for 2^−ΔΔCT^ calculation.

**Table 2:** SYBR-Green primers for real time PCR.

*name*	*forward primer*	*reverse primer*
18S	5′GCAATTATTCCCCATGAACG	5′GGCCTCACTAAACCATCCAA
Syncytin-1	5′ATGGAGCCCAAGATGCAG	5′AGATCGTGGGCTAGCAG
DNMT1	5′TAAAGCCTGCAAGGACATGGT	5′TGGGTGACGGCATCTCTGG
DNMT3a	5′CGTGGCAAGGAGGAGCG	5′TCTGAGGCGCCTGAGTCC
DNMT3b	5′CACAGGCCTTCCCCACGT	5′CGTCTGTGAGGTCGATGGTAA
MBD1	5′GATCTCACCCTCTTCGACTTC	5′TCCGAGTCTTGGCTGGCCT
MBD2	5′GCTGTTTGGCTTAACACATCTCA	5′CAAGATGTCTGCCATCAGTGC
MBD3	5′GGTCAAGAGCGACCCGCA	5′CAGGGTGCCCGCTCACC
MBD4	5′AGATGTGTCAGAACTTCTTAAACCT	5′TCATTGACACAAAAAATTCGGTAAGA
MeCP2	5′GCTCTGCTGGGAAGTATGATG	5′GTGAAGTCAAAATCATTAGGGTCC
LSH	5′GAGTACACGAGCTGGTGGCCTGGG	5′CTGGGCCTGAAGATCCGACTGGGG

### Genomic DNA Extraction

Cultivated CTs or 50–100 mg placental tissues were minced using a Micro-Dismembranator (Braun Biotech, Sartorius AG) and lysed in 3 ml cell-lysis-buffer overnight at 50°C. After incubation with RNaseA DNA was extracted by Phenol-Chloroform-Isoamylalcohol (Sigma) isolation, precipitated with 1.2M NH_4_Ac in isopropanol and dissolved in 0.01% DEPC water.

### Bisulfite Treatment of Genomic DNA and Bisulfite Sequencing

Bisulfite treatment of genomic DNA was performed with the EpiTect Bisulfite Kit (Qiagen) according to the manufacturers’ instructions. Specific fragments of the 5′LTR of ERVW-1 on chromosome 7q21.2 were amplified with the forward primers (Syn1UF 5′AGGATTAGTTGGATTTTTTAGGTTGA3′; Syn1MF 5′TAGGATTAGTTGGATTTTTTAGGT C3′) were set within the 5′LTR over the first CpG (methylated (M) and unmethylated (U) primer version) and the reverse primer (Syn1R 5′CCCAAATAACCTCACACCTA3′ downstream of the 5′LTR (without any CpG). For the correct distribution of the methylated and unmethylated fragments within the DNA probe PCR was performed with the 2 forward and 1 reverse primer with 2.5U HotStarTaq (Qiagen) and fragments were cloned into the StrataClone vector (pSC-A-amp/kan). For each DNA probe a minimum of 10 different clones were sequenced with the ABI3730 DNA Analyser (Applied Biosystems) and sequences were analysed with the Geneious 4.6.4 software. Pyrosequencing was done with the PyroMark Q24 (Qiagen). Fragments for the two CpGs within the TSE were amplified (TSE_TF 5′TGTGGTTATGTGATATAGTTTTGG_biotinylated and TSE_BR 5′CGACCTAAAAAATCCAACTAATCC) with the EpiTect HRM PCR Master Mix (Qiagen) and 50 ng bisulfite treated DNA. Sequencing reaction was setup with the PyroMark Gold Q24 Reagents (Qiagen) and the sequencing primer (TSE_seq 5′ATTACAAAATAATTACTATATCTCC). Data were analyzed with the PyroMark Q24 Software.

### Determination of Fusion Index (FI) by Membrane Staining

Cell cultures at day 3 were analysed microscopically for cell fusions using wheat germ agglutinin (Alexa594) plasma membrane staining along with the nuclear stain Hoechst 33342 (both Molecular Probes) [16], [36], [50]. Ten different visual fields from each culture were analyzed to determine fusion-index (FI) and number of nuclei/SCT by two independent researchers [16]. Analysis was performed by microscopy (Olympus BX51). Images were acquired with Olympus color-view and applying the program Cell-F. Images were further processed with Photoshop CS3.

### Luciferase Assay

The 5′LTR of the ERVW-1 promoter was cloned into the luciferase plasmid pGL3basic (Promega) [55]. Luciferase assays (Roche) along with methylated and unmethylated vector and AZA were done in parallel with independent transfections of BeWo cells using the JetPEI transfection reagent (PeqLab) then analyzed 48 hr post-transfection (1 µg 5′LTR plasmids for the promoter assay and a transfection control with 1 µg of the β-Galactosidase expression-plasmid pSVβGal (Promega)). Statistics were performed with a minimum of 5 measurements per plasmid. Values were normalized to pGL3basic and transfection control.

### In vitro Methylation Assay

The in vitro methylation of the 5′LTR-pGL3 plasmid was performed with the CpG-methyltransferase-kit (New England Biolabs) according to the manufacturers’ instructions.

### Statistical Analysis

The nonparametric Mann–Whitney-U-test for independent samples was performed using SPSS 18.0.0. (SPSS, Inc.). For all tests, a P<0.05 was considered as statistically significant. For each mean value, a standard error of the mean (sem) was calculated using SPSS 18.0.0.

## Results

### Bisulfite Sequencing of the ERVW-1 Promoter Region

**Figure 1:**
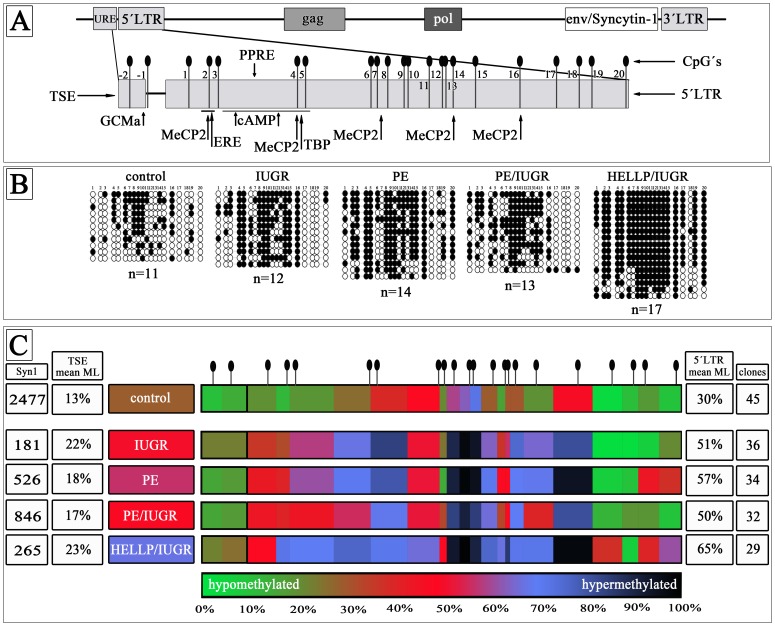
Methylation Profile of the entire promoter of ERVW-1 in isolated trophoblasts. A) Locus of Syncytin-1 on chromosome 7q21.2 within the ERVW-1 gene. Black needles represent the single CpG sides in the promoter. Arrows mark binding sites for transcription factors ore methyl binding proteins B) All sequenced clones of control (n = 11), IUGR (n = 12), PE (n = 14), PE/IUGR (n = 13) and HELLP/IUGR (n = 17) from one placental trophoblast isolation of each group. White circles represent unmethylated CpGs and black circles methylated CpGs. C) MethylationHeatMap of the promoter region. Mean of three different placentae of each group. Mean of single CpGs are color-coded. From green unmethylated (0%) over red (50% methylated) to black (100% methylated). meanML → mean methylation level of the entire promoter; clones → total analysed clones of each group; Syn1 → mean Syncytin-1 gene expression in mol/ng_cDNA._

In order to identify methylated CpGs within the 5′LTR of ERVW-1 on Chromosome 7q21.2 genomic DNA was bisulfite treated and cloned into the pSC-A-amp/kan sequencing vector. For a trophoblast specific methylation profile of the 5′LTR of ERVW-1 we isolated genomic DNA from purified CTs. The mean methylation level (ML) was 31% in isolated control CTs (n = 3). All pathological CTs showed a significant hypermethylation from 50% in PE/IUGR (n = 3) over 51% in IUGR (n = 3) and 57% in PE (n = 3) to 65% HELLP/IUGR (n = 2) (Fig. 1; Table S1). In all pathological CTs 11 out of 20 CpGs were significantly hypermethylated (CpG2–5,8,9,11,12,14–16). CpG6 and 18 were identically methylated in all examined CTs. In IUGR only CpG10 was additionally hypermethylated (94%) even though CpG10 was methylated in control CTs (73%) (Fig. 1; Table S1). CTs from PE placentae showed also a significant higher ML for CpG1,10,19 and 20 whereas in PE/IUGR CpG1 and 10 were hypermethylated but CpG19 and 20 were unchanged (Fig. 1; Table S1). CTs from HELLP/IUGR had the most hypermethylated CpGs compared to control CTs, only CpG6 and 18 showed no differences to control CTs (Fig. 1; Table S1). These results suggest that reduced Syncytin-1 gene expression in pathological CTs could be due to hypermethylation of the 5′LTR promoter region of ERVW-1 on chromosome 7q21.2. Further we analysed the 2 CpGs (CpG −1, −2) within the TSE upstream of the 5′LTR of ERVW-1 (Fig. 1A). For this methylation profile we used the CpG-method by pyrosequencing. No significant changes between control and pathological CTs could be shown; however CTs isolated from HELLP/IUGR placentae showed a trend towards a higher methylated profile (23%) compared to control (13%) (Fig. 1; Table S1).

### Effects of AZA and TSA Treatment on Trophoblast-like Cell Lines

**Figure 2:**
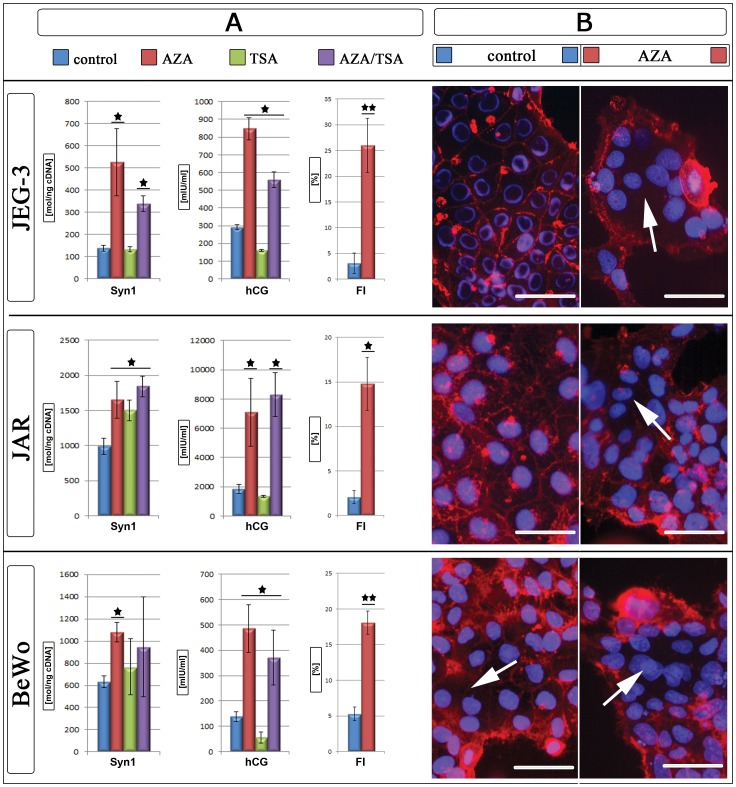
AZA and TSA treatment of trophoblast-like cell lines. A) Expression data of Syncytin-1 in molecules per ng cDNA [mol/ng_cDNA_] and hCG [mlU/ml] for control (blue), AZA (red), TSA (green) and AZA/TSA (violet) treated cell lines and Fusion index [%] of control (blue) and AZA (red) treated cultures. B) For membrane stains of control (blue) and AZA (red) treated cell lines cell membranes were stained with wheat germ agglutinin Alexa594 (red) and nuclei with Hoechst 33342 (blue). White arrows mark syncytia. *P≤0.05; **P≤0.005; bars represent 50 µm.

To further determine, if Syncytin-1 is regulated by DNA methylation, we induced gene expression by the use of AZA causing genomic DNA demethylation. For our experiments three trophoblast-like cell lines JEG-3, JAR and BeWo were used. After three days gene expression of Syncytin-1 as well as hCG levels were determined in the cell culture supernatant. Bisulfite sequencing of these cell lines resulted in a mean ML of the 5′LTR of ERVW-1 of 51% in JEG-3, 53% in JAR and 39% in BeWo. Expression of the trophoblast differentiation marker hCG increased significantly in all three cell lines after AZA treatment (Fig. 2). In all cell lines Syncytin-1 increased gene expression significantly after AZA treatment (Fig. 2).

DNA methylation is linked to histone acetylation and chromatin condensation, which is performed by recruitment of histone deacetylases (HDAC). Trichostatin A (TSA), an inhibitor of HDAC can block histone deacetylation. HCG expression in the supernatant of TSA treated cell lines was down regulated (JEG-3 1.8-fold; JAR 1.4-fold, BeWo 2.4-fold) (Fig. 2). Even though lower hCG amounts in JAR were detected, Syncytin-1 gene expression increased significantly 1.5-fold compared to controls (Fig. 2). For JEG-3 and BeWo no changes occurred for Syncytin-1 after TSA treatment (Fig. 2).

In addition, we examined the effects of a combined treatment of AZA and TSA. In supernatants of all cell lines the hCG expression increased significantly with AZA/TSA compared to untreated controls after 72 hr (Fig. 2). Syncytin-1 expression increased in all cell lines with AZA/TSA (JEG-3 2.5-fold and JAR 1.9-fold), but for BeWo (1.5-fold) not significant (Fig. 2). In comparison to the AZA and TSA treatment alone no additive effect could be shown with the combined treatment of AZA and TSA in the trophoblast-like cell lines. In return there was a decreased gene expression when the cells were treated with AZA and TSA together in comparison to the individual treatment of both drugs (Fig. 2). Further we analysed, if the higher Syncytin-1 gene expression was due to an overexpression of the coding 3.1 kb and not only of the 8.0 kb Syncytin-1 transcript. In all analysed cell lines and treatments the 3.1 kb transcript was more than 2-fold higher expressed than the 8.0 kb transcript (data not shown).

### Effects of AZA Treatment on Fusion Index

Our gene expression analysis showed that JEG-3, JAR and BeWo are sensitive to an AZA treatment which resulted in an up-regulation of Syncytin-1 after a 72 hr incubation period. The question was, if this up-regulation of the fusogenic Syncytin-1 was also affecting the fusion ability of the trophoblast-like cell lines. Therefore we performed a fusion assay and determined the fusion index (FI) with and without AZA treatment. In all cell lines only a few multinucleated cells could be detected in the untreated control: JEG-3 3.1%; JAR 2.1% and BeWo 5.3% (Fig. 2). Especially for JEG-3 and JAR no syncytia with more than 3 nuclei could be found, whereas BeWo had up to 6 nuclei in fused cells. After AZA treatment FI raised significantly in all cell lines up to 26.0% for JEG-3, 14.8% for JAR and 18.1% for BeWo (Fig. 2). Fusions with up to 11 and 17 nuclei could be found in JEG-3 and BeWo cultures, respectively.

### Hypermethylation of the 5′LTR Inhibited Promoter Activity of ERVW-1

**Figure 3:**
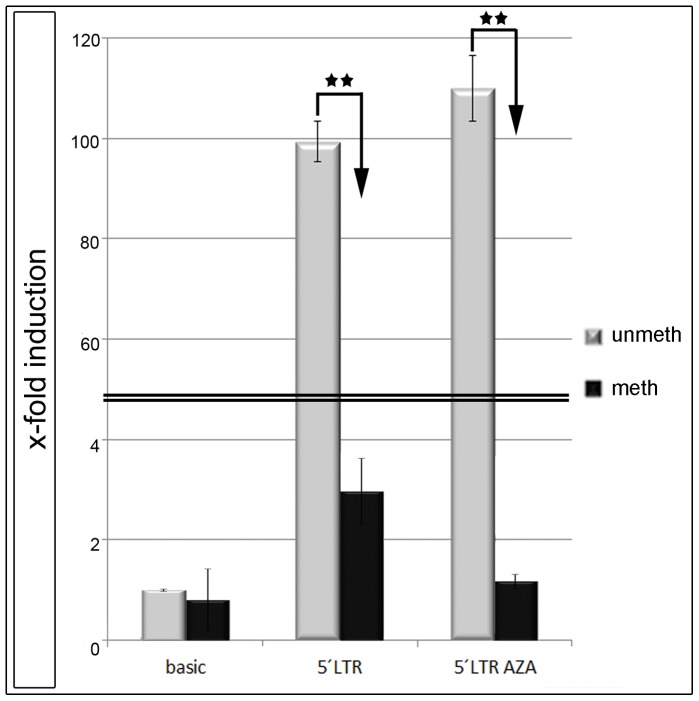
Luciferase Assay. The 5′LTR of ERVW-1 was cloned in the pGL3-basic vector and transfected in BeWo cells. 48 hr post transfection cells were analysed. Luciferase activity of the pGL3-basic vector was set to 1. All experiments were repeated at least five times. Histographs show fold-induction to pGL3-basic. (**P<0.005).

We could show that the amount of methylated CpGs within entire promoter of ERVW-1 correlated with a reduced gene expression of Syncytin-1 in isolated trophoblasts of control and pathological placentae. A demethylation with AZA resulted in an up-regulation of Syncytin-1 in trophoblast-like cell lines. In order to confirm these findings we cloned the 5′LTR of ERVW-1 into the pGL3-basic vector, which had only a basal luciferase activity after transfection. With this vector construct (5′LTR-pGL3) we were able to analyse the influence of DNA methylation and AZA treatment on the 5′LTR promoter activity in BeWo cells. Unmethylated 5′LTR raised the luciferase activity up to 92.5-fold compared to the pGL3-basic vector (Fig. 3). In vitro methylation of the 5′LTR-pGL3 resulted in a 2.2-fold up-regulation of luciferase activity, or a 42.0-fold decline compared to the unmethylated 5′LTR-pGL3 vector (Fig. 3). Further we wanted to know, whether the up-regulation of Syncytin-1 was only a secondary effect of the AZA treatment by an activation of specific transcription factors, or if it was directly linked to demethylation. Therefore we transfected the unmethylated 5′LTR-pGL3 vector in BeWo cells and treated them with AZA over 72 hr in parallel. Compared to untreated cell culture no changes in luciferase activity were detectable (Fig. 3).

### Aberrant DNMT and MBD Gene Expression

**Figure 4:**
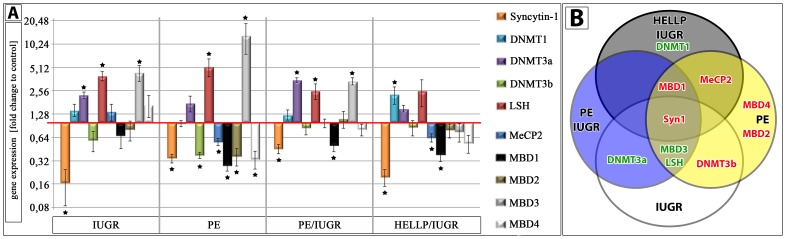
Gene expression profile of Syncytin-1 and methyl-binding proteins in isolated trophoblasts. A) Histograph showing fold-change differences to gene expression profile (2^−ΔΔCT^) of control trophoblasts of IUGR, PE, PE/IUGR and HELLP/IUGR (each n = 7). Red line marks gene expression profile of control trophoblasts set to 1. (*P≤0.05). B) Venn diagram showing for each placental syndrome the statistical significant different expressed genes compared to control VCTs. The intersection of all four placental syndromes represents Syncytin-1.

To further analyse the reason for the hypermethylation of the 5′LTR of ERVW-1 in pathological CTs we determined the gene expression profile of DNA-methyltransferases (DNMT1, 3a, 3b, LSH) and methyl binding proteins (MBD1-4, MeCP2) by sqPCR. In all pathological CTs Syncytin-1 decreased significantly to controls (n = 7) (Fig. 4A). All syndromes showed a specifically changed gene expression profile. In IUGR CTs DNMT3a, LSH and MBD3 were up-regulated and DNMT3b down-regulated. In PE only LSH and MBD3 were over expressed and DNMT3b, MeCP2, MBD1, MBD2 and MBD4 under expressed. DNMT3a, LSH and MBD3 of PE/IUGR were up- and only MBD1 down-regulated, and finally in HELLP/IUGR only DNMT1 was increased, MeCP2 and MBD1 decreased (Fig. 4A/B).

## Discussion

### Hypermethylation of the Entire Promoter of ERVW-1 Correlated with Reduced Syncytin-1 Expression in Pathological Trophoblasts

Recently Gao and his group found that Syncytin-1 was increased in discordant twins small for gestational age (SGA) and that this up-regulation was due to a hypomethylation of the ERVW-1 promoter [56]. We asked the question, if reduced Syncytin-1 expression in IUGR, PE and HELLP which we demonstrated [16], [50] could be due to an aberrant DNA methylation of the promoter region of this gene. The mean ML in term control fractionated VCTs were identical to the findings of Gimenez et al. and Macaulay at al. [49], [57]. In all pathological fractionated VCTs the mean ML rose from 29% in controls to 47% to 64%. These findings of a hypermethylated promoter region of ERVW-1 went along with a reduced Syncytin-1 gene (Fig. 1; Table S1), protein expression and FI as shown in previous publications [16], [50], [55]. HELLP/IUGR VCTs had the highest hypermethylation with 64% ML. Figure 1 shows that especially the region upstream of the TATA box (CpG1 to CpG4) was hypomethylated in control VCTs (25%). Important transcription factors like TATA box binding protein (TBP), Estrogen-Receptor, p-CREB and PPARγ/RXRα heterodimer can bind to this promoter region [36], [38], [55]. Prudhomme and colleagues identified an upstream regulatory element (URE) which is active in placental cells. Furthermore they showed within this URE a trophoblast-specific enhancer (TSE) containing a GCMa binding site and two potential methylation sites [38]. Interestingly no significant changes of methylation occurred between control and pathological CTs at the TSE (Fig. 1, Table S1). These results indicate that the TSE is important for the basal promoter activity of ERVW-1 and is somehow protected from a hypermethylation in trophoblastic cells. Proteins with a methyl-binding-domain (MBD) can recognise methylated promoter regions [7]. Especially MeCP2 preferentially bind single CpGs near a consensus sequence (A/T≥4) [58]. Within the promoter of the murine receptor activator of nuclear factor –B ligand (RANKL) a specific binding of MeCP2 to a methylated CpG, 3 bases upstream of the TATA box, was shown [59]. Exactly the same conditions exist for CpG4 within the ERVW-1 promoter. Besides CpG4, four other CpGs (2, 7, 14 and 16) include the same consensus sequences for MeCP2-binding, however CpG16 had the highest homology. In case of hypermethylation MeCP2 is competing against other transcription factors (ER, GATA, SP1, AP-1, AP-3), which could bind to this region. Furthermore bound MeCP2 mediates histone deacetylation and chromatin condensation [60], [61]. Therefore an aberrant hypermethylation of the 5′LTR of ERVW-1 could contribute to the decreased expression levels of Syncytin-1 in pregnancy associated diseases.

### Demethylation with AZA Increased Syncytin-1 Expression and Fusion in Cell Lines

In order to verify our hypotheses that a hypomethylated ERVW-1 promoter increases Syncytin-1 expression, the trophoblast-like cell lines JEG3, JAR and BeWo were treated with AZA. This cytosine analogue is integrated as frequently as the endogenous cytosine into DNA by DNA polymerase but no methyl-group is addable. Finally a treatment with AZA causes a genomic demethylation in proliferating cells [62]. After a 3 day period of AZA treatment in all trophoblast-like cell lines Syncytin-1, hCG expression and FI significantly increased indicating an epigenetically regulation of this gene. This was further verified by luciferase assays in BeWo cells with the 5′LTR of ERVW-1. The unmethylated 5′LTR induced a strong luciferase activity, whereas a transfected methylated 5′LTR showed no luciferase expression. An additional treatment with AZA had no stimulatory effect on the luciferase activity. This indicated that the effect was directly linked to the methylation pattern of Syncytin-1 promoter. Rahnama et al. (2006) showed that an AZA treatment of BeWo cells inhibited migration and invasion ability in this cell line. We could now further show, that this demethylation induced cell fusion in BeWo up to 3.4-fold [63]. This finding now raises the question about the methylation profile of EVT. Malassiné et al. could show that EVTs have a 15-fold lower expression of Syncytin-1 compared to VCT [64]. Strick et al. found that the ratio between Syncytin-1 and TGF-ß is regulating cell fusion. TGF-ß induced proliferation whereas Syncytin-1 activated the fusion pathway [36]. We now propose that Syncytin-1 activation by a hypomethylated 5′LTR is increasing cell fusion in contrast to hypermethylation which is decreasing fusion and, along with a reduced Syncytin-1 expression, increasing invasion. Blocking HDACs with TSA in cell lines had no effect on Syncytin-1 expression for JEG-3 and BeWo, but significantly down-regulated hCG. For JAR a 1.5-fold up-regulation could be shown, whereas hCG was unchanged compared to control. Only for JAR a minor additive effect could be seen with the combined use of AZA and TSA for Syncytin-1 and hCG. Chuang et al. could show that treatment with TSA up-regulated the placenta specific transcription factor GCMa [65]. Upstream of the 5′LTR of ERVW-1 two GCMa binding sites were identified which can increase Syncytin-1 expression and cell fusion in JEG3 and BeWo [66]. This could be the reason for Syncytin-1 overexpression in JAR cells after TSA treatment. On the other side an inhibition of proliferation by TSA could also be shown [67]. Furthermore we propose that the inhibition of gene expression by hypermethylation of the ERVW-1 promoter is not basically due to a recruitment of HDACs and chromatin condensation as indicated by the ineffective TSA treatment in BeWo and JEG-3 cells. We believe that the inhibition of Syncytin-1 gene expression is mediated by competition of methyl-binding-proteins, like MeCP2 with transcription factors and proteins necessary for the RNA transcription machinery. One example for the hypothesis of competing binding factors at CpGs is the work by Curradi et al (2002), who showed that transcriptional activators compete with methylation-specific repressors and vice versa even in vivo [68].

### Changes of the DNA-methyltransferase Profile could cause Genomic Hypermethylation

Epigenetic marks have to be set by DNA-methyltransferases (DNMTs, LSH) and these marks have to be recognised by proteins with a methyl-binding-domain (MBDs) and mediate gene silencing through e.g. histone deacetylation by recruitment of HDACs [7]. We found significant changes in the expression profile of these genes in pathological VCTs compared to control VCTs. Novakovic et al. (2010) found a specific hypermethylation of the maintenance DNA methyltransferase DNMT1, which was required for a genomic hypomethylation [69]. This could explain our findings in VCTs from HELLP/IUGR. DNMT1 expression was significantly up-regulated indicating a genomic hypermethylation in these placentae. Rahnama et al. (2006) could show that the promoter activity of Plakoglobin and E-cadherin was reverse regulated by DNMT3a and -3b [63]. This could also be the reason for low Syncytin-1 levels in VCTs of IUGR, PE and PE/IUGR where an increased DNMT3a expression may mediate an epigenetic hypermethylation in the promoter region. The function of LSH during human placentogenesis is completely unknown to date, but our results indicate a role of LSH during aberrant DNA methylation. Tao et al. could show that LSH mediated a RNA-polymerase II stalling at HOX gene promoter sequences [70]. Further they found that decreased LSH was associated with reduced DNMT3b binding to promoter regions in breast cancer [71]. In return increased LSH would mediate DNMT3b binding and hypermethylation what could be the reason for the aberrant epigenetic in the pathological CTs. Huang et al. (2004) found that LSH is an epigenetic guardian of repetitive elements [72]. Gene expression from brain and liver tissue of LSH^−/−^ embryos showed that almost two-third of aberrant expressed genes contained mainly retroviral LTRs indicating that LSH is regulating preferentially repeat elements [72]. Therefore, hypermethylated 5′LTRs in the pathological VCTs could be linked especially to overexpressed LSH. This would also explain our previous findings in isolated VCTs of IUGR placentae, where we found a decreased gene expression of 4 other ERV env genes (Syncytin-2, Erv3, EnvV1 and EnvV2) [16].

In PE we could show a decrease of the tumor suppressor gene MBD4. This protein plays multiple functions in cellular processes including DNA mismatch repair, apoptosis and transcriptional repression [73]. Especially at CpG sites MBD4 repairs G:T and G:U mismatches from spontaneous deamination events of methylated and unmethylated cytosines [74]. A lowered mismatch repair rate could cause an accumulation of mutations with unpredictable effects not only to the placenta but also to the mother causing PE. The work of Yildirim et al. (2011) showed that a MBD3 knockdown preferentially affected expression of 5-hydroxymethylcytosine (5 hmC) marked genes in embryonic stem cells [75]. Even though we cannot distinguish between 5 hmC and 5-methylcytosine, MBD3 overexpression in IUGR, PE and PE/IUGR placentae could explain lowered Syncytin-1 levels. Finally Trejbalová et al. (2011) showed that repression of Syncytin-1 and Syncytin-2 was also linked to histone H3 lysine 9 (H3K9) trimethylation in HeLa compared to BeWo cells [76]. Their results showed that different levels of an epigenetic regulation could be responsible for the expression or repression of Syncytin-1. If low Syncytin-1 levels in pathological VCTs are also linked to an aberrant H3K9 methylation profile this has to be evaluated in additional studies.

### Conclusions

We could show that reduced Syncytin-1 expression in placental syndromes was due to an epigenetic hypermethylation of the entire promoter region of ERVW-1. We propose that a promoter hypermethylation occurs in these pathological placentae due to the fact that DNA-methyltransferases, which are responsible for the setting of these epigenetic marks, are overexpressed.

## Supporting Information

Table S1 **Methylation pattern of isolated trophoblasts.**
Methylation pattern of all 22 CpGs of isolated trophoblasts from control (n = 3), IUGR (n = 3), PE (n = 3), PE/IUGR (n = 3) and HELLP/IUGR (n = 2) placentae. CpG (−1) and CpG (−2) are located within the TSE and CpG 1 to 20 within the 5′LTR (Fig. 1A). Syncytin-1 gene expression is shown in molecules/ng_cDNA_. p<0.05 → statistically significant.
(DOC)
