# Correction: Reduced Syncytin-1 Expression Levels in Placental Syndromes Correlates with Epigenetic Hypermethylation of the ERVW-1 Promoter Region

**DOI:** 10.1371/journal.pone.0107215

**Published:** 2014-08-29

**Authors:** The PLOS ONE Staff

An earlier version of Table 1 incorrectly appears in the article. Please see the correct Table 1 here.

**Table 1:** Clinical data.

	control n = 3		IUGR n = 3		PE n = 3		PE/IUGR n = 3		HELLP/IUGR n = 2	
	mean	sem	mean	sem	mean	sem	mean	sem	mean	sem
**gestational age**	37.67	±0.33	36.33	±2.33	37.33	±1.20	35.67	±2.96	37.50	±0.50
**gravida**	1.67	±0.33	1.33	±0.33	1.33	±0.33	1.33	±0.33	1.00	±0.00
**para**	0.33	±0.33	0.33	±0.33	0.33	±0.33	0.33	±0.33	0.00	±0.00
**blood pressure**	113.33	±8.82	136.67	±6.67	178.33	±1.67	180.00	±12.58	170.00	±10.00
	61.67	±1.67	78.67	±6.84	103.33	±3.33	108.00	±11.79	110.00	±10.00
**protein**	n.d.		n.d.		7,971.33	±2,242.40	4,360.67	±2,941.75	6,536.00	±303.00
**GOT**	n.d.		n.d.		36.33	±6.84	69.33	±37.84	518.50	±187.50
**GPT**	n.d.		n.d.		24.67	±2.96	62.00	±48.00	367.50	±127.50
**LDH**	n.d.		n.d.		242.50	±31.44	277.33	±26.03	1076.00	±163.00
**platelets**	245.67	±77.45	210.67	±19.54	254.67	±34.64	228.00	±33.42	90.50	±8.50
**weight child**	3,346.67	±331.93	1,743.33	±326.82	2,900.00	±315.33	2,208.67	±899.59	2,105.00	±295.00
**height child**	50.67	±1.86	40.33	±3.18	48.33	±1.67	42.33	±6.12	45.00	±3.00

GOT:Glutamate-Oxaloacetate-Transaminase; GPT: Glutamate-Pyruvate-Transaminase; LDH: Lactate dehydrogenase.
