# Supplementary material for: Reduced Syncytin-1 Expression Levels in Placental Syndromes Correlates with Epigenetic Hypermethylation of the ERVW-1 Promoter Region
Source: PLoS One. 2013 Feb 14;8(2):e56145. doi: 10.1371/journal.pone.0056145 (PMC3573012; doi:10.1371/journal.pone.0056145)
Supplement: Table S1 — Methylation pattern of isolated trophoblasts. Methylation pattern of all 22 CpGs of isolated trophoblasts from control (n = 3), IUGR (n = 3), PE (n = 3), PE/IUGR (n = 3) and HELLP/IUGR (n = 2) placentae. CpG (−1) and CpG (−2) are located within the TSE and CpG 1 to 20 within the 5′LTR (Fig. 1A). Syncytin-1 gene expression is shown in molecules/ngcDNA. p<0.05 → statistically significant. (DOC) [file pone.0056145.s001.doc]

**Supplemental Table S1:** Methylation pattern of isolated trophoblasts

|  | control | | IUGR | | | PE | | | PE/IUGR | | | HELLP | | |
| --- | --- | --- | --- | --- | --- | --- | --- | --- | --- | --- | --- | --- | --- | --- |
|  | **mean** | sem | **mean** | sem | p-value | **mean** | sem | p-value | **mean** | sem | p-value | **mean** | sem | p-value |
| CpG (-1) | **14.67%** | 1.45% | **22.00%** | 4.73% | 0.1840 | **19.00%** | 2.52% | 0.1840 | **18.33%** | 1.33% | 0.1046 | **24.50%** | 3.50% | 0.0833 |
| CpG (-2) | **12.00%** | 1.53% | **21.33%** | 6.17% | 0.2752 | **16.33%** | 2.33% | 0.1046 | **16.00%** | 3.22% | 0.2752 | **22.00%** | 4.00% | 0.0833 |
| CpG1 | **22.22%** | 6.27% | **36.11%** | 8.12% | 0.1710 | **44.12%** | 8.64% | 0.0395 | **43.75%** | 8.91% | 0.0458 | **48.28%** | 9.44% | 0.0203 |
| CpG2 | **13.33%** | 5.12% | **33.33%** | 7.97% | 0.0325 | **41.18%** | 8.57% | 0.0051 | **40.63%** | 8.82% | 0.0065 | **72.41%** | 8.45% | <0.0001 |
| CpG3 | **17.78%** | 5.76% | **58.33%** | 8.33% | 0.0002 | **61.76%** | 8.46% | 0.0001 | **43.75%** | 8.91% | 0.0135 | **68.97%** | 8.74% | <0.0001 |
| CpG4 | **26.67%** | 6.67% | **66.67%** | 7.97% | 0.0003 | **70.59%** | 7.93% | 0.0001 | **56.25%** | 8.91% | 0.0092 | **75.86%** | 8.09% | <0.0001 |
| CpG5 | **40.00%** | 7.39% | **83.33%** | 6.30% | 0.0001 | **79.41%** | 7.04% | 0.0005 | **68.75%** | 8.32% | 0.0134 | **72.41%** | 8.45% | 0.0068 |
| CpG6 | **51.11%** | 7.54% | **52.78%** | 8.44% | 0.8821 | **52.94%** | 8.69% | 0.8727 | **53.13%** | 8.96% | 0.8625 | **65.52%** | 8.98% | 0.2252 |
| CpG7 | **22.22%** | 6.27% | **25.00%** | 7.32% | 0.7708 | **14.71%** | 6.17% | 0.4020 | **34.38%** | 8.53% | 0.2411 | **48.28%** | 9.44% | 0.0203 |
| CpG8 | **57.78%** | 7.45% | **88.89%** | 5.31% | 0.0022 | **91.18%** | 4.94% | 0.0011 | **84.38%** | 6.52% | 0.0136 | **93.10%** | 4.79% | 0.0011 |
| CpG9 | **64.44%** | 7.22% | **97.22%** | 2.78% | 0.0003 | **97.06%** | 2.94% | 0.0005 | **90.63%** | 5.24% | 0.0091 | **100.00%** | 0.00% | 0.0003 |
| CpG10 | **73.33%** | 6.67% | **94.44%** | 3.87% | 0.0131 | **94.12%** | 4.10% | 0.0173 | **84.38%** | 6.52% | 0.2527 | **89.66%** | 5.76% | 0.0904 |
| CpG11 | **28.89%** | 6.83% | **63.89%** | 8.12% | 0.0017 | **70.59%** | 7.93% | 0.0003 | **65.63%** | 8.53% | 0.0015 | **75.86%** | 8.09% | 0.0001 |
| CpG12 | **15.56%** | 5.46% | **36.11%** | 8.12% | 0.0341 | **47.06%** | 8.69% | 0.0024 | **34.38%** | 8.53% | 0.0561 | **65.52%** | 8.98% | <0.0001 |
| CpG13 | **31.11%** | 6.98% | **55.56%** | 8.40% | 0.0277 | **47.06%** | 8.69% | 0.1507 | **50.00%** | 8.98% | 0.0961 | **86.21%** | 6.52% | <0.0001 |
| CpG14 | **31.11%** | 6.98% | **75.00%** | 7.32% | 0.0001 | **67.65%** | 8.14% | 0.0014 | **68.75%** | 8.32% | 0.0012 | **72.41%** | 8.45% | 0.0006 |
| CpG15 | **20.00%** | 6.03% | **63.89%** | 8.12% | 0.0001 | **67.65%** | 8.14% | 0.0000 | **40.63%** | 8.82% | 0.0498 | **72.41%** | 8.45% | <0.0001 |
| CpG16 | **51.11%** | 7.54% | **80.56%** | 6.69% | 0.0063 | **94.12%** | 4.10% | 0.0000 | **81.25%** | 7.01% | 0.0071 | **96.55%** | 3.45% | <0.0001 |
| CpG17 | **4.44%** | 3.11% | **2.78%** | 2.78% | 0.6949 | **588%** | 4.10% | 0.7743 | **12.50%** | 5.94% | 0.1966 | **37.93%** | 9.17% | 0.0002 |
| CpG18 | **11.11%** | 4.74% | **2.78%** | 2.78% | 0.1573 | **8.82%** | 4.94% | 0.7402 | **18.75%** | 7.01% | 0.3483 | **6.90%** | 4.79% | 0.5481 |
| CpG19 | **17.78%** | 5.76% | **5.56%** | 3.87% | 0.0987 | **44.12%** | 8.64% | 0.0112 | **18.75%** | 7.01% | 0.9138 | **41.38%** | 9.31% | 0.0266 |
| CpG20 | **11.11%** | 4.74% | **22.22%** | 7.03% | 0.1785 | **38.24%** | 8.46% | 0.0047 | **9.38%** | 5.24% | 0.8069 | **62.07%** | 9.17% | <0.0001 |
| CpGs TSE | **13.33%** | 1.12% | **21.67%** | 3.48% | 0.0649 | **17.67%** | 1.65% | 0.0649 | **17.17%** | 1.64% | 0.0931 | **23.25%** | 2.29% | 0.0095 |
| CpGs 5´LTR | **30.53%** | 4,39% | **50.70%** | 6.64% | 0.0224 | **56.93%** | 6.19% | 0.0035 | **50.06%** | 5.56% | 0.0122 | **65.36%** | 5.26% | <0.0001 |
| CpGs all | **28.98%** | 4.1% | **49.39%** | 6.46% | 0.0166 | **53.34%** | 6.13% | 0.0069 | **47.05%** | 5,45% | 0.0161 | **63.53%** | 5.34% | <0.0001 |
| Syncytin1 | **2477.56** | 43.91 | **181.25** | 18.01 | 0.0500 | **526.01** | 38.05 | 0.0500 | **845.87** | 115.58 | 0.0500 | **264.55** | 150.12 | 0.0500 |

Methylation pattern of all 22 CpGs of isolated trophoblasts from control (n=3), IUGR (n=3), PE (n=3), PE/IUGR (n=3) and HELLP/IUGR (n=2) placentae. CpG (-1) and CpG (-2) are located within the TSE and CpG 1 to 20 within the 5´LTR (Fig.1A). Syncytin-1 gene expression is shown in molecules/ngcDNA. p<0.05 → statistically significant.
